# Super-mini percutaneous nephrolithotomy (SMP) vs retrograde intrarenal surgery (RIRS) in the management of renal calculi ≤ 2 cm: a propensity matched study

**DOI:** 10.1007/s00345-021-03860-w

**Published:** 2021-11-12

**Authors:** Sunil Bhaskara Pillai, Arun Chawla, Jean de la Rosette, Pilar Laguna, Rajsekhar Guddeti, Suraj Jayadeva Reddy, Ravindra Sabnis, Arvind Ganpule, Mahesh Desai, Aditya Parikh

**Affiliations:** 1 Department of Urology and Renal Transplant, Kasturba Medical College, Manipal Academy of Higher Education (MAHE), Manipal, Karnataka India; 2 Istanbul Medipol Mega University Hospital, Istanbul, Turkey; 3 Muljibhai Patel Urological Hospital, Nadiad, Gujarat India

**Keywords:** RIRS, SMP, PCNL, Renal stones, Miniaturization, Flexible

## Abstract

**Objective:**

To compare the effectiveness and safety of Super-Mini PCNL (SMP) and Retrograde Intrarenal Surgery (RIRS) in the management of renal calculi ≤ 2 cm.

**Patients and methods:**

A prospective, inter-institutional, observational study of patients presenting with renal calculi ≤ 2 cm. Patients underwent either SMP (Group 1) or RIRS (Group 2) and were performed by 2 experienced high-volume surgeons.

**Results:**

Between September 2018 and April 2019, 593 patients underwent PCNL and 239 patients had RIRS in two tertiary centers. Among them, 149 patients were included for the final analysis after propensity-score matching out of which 75 patients underwent SMP in one center and 74 patients underwent RIRS in the other. The stone-free rate (SFR) was statistically significantly higher in Group 1 on POD-1 (98.66% vs. 89.19%; *p* = 0.015), and was still higher in Group 1 on POD-30 (98.66% vs. 93.24%, *p* = 0.092) SFR on both POD-1 and POD-30 for lower pole calculi was higher in Group 1 (100 vs. 82.61%, *p* = 0.047 and 100 vs 92.61% *p* = 0.171). The mean (SD) operative time was significantly shorter in Group 1 at 36.43 min (14.07) vs 51.15 (17.95) mins (*p* < 0.0001). The mean hemoglobin drop was significantly less in Group 1 (0.31 vs 0.53 gm%; *p* = 0.020). There were more Clavien–Dindo complications in Group 2 (*p* = 0.021). The mean VAS pain score was significantly less in Group 2 at 6 and 12 h postoperatively (2.52 vs 3.67, 1.85 vs 2.40, respectively: *p* < 0.0001), whereas the mean VAS pain score was significantly less in Group 1 at 24 h postoperatively (0.31 vs 1.01, *p* < 0.0001). The mean hospital stay was significantly shorter in Group 1 (28.37 vs 45.70 h; *p* < 0.0001).

**Conclusion:**

SMP has significantly lower operative times, complication rates, shorter hospital stay, with higher stone-free rates compared to RIRS. SMP is associated with more early post-operative pain though.

## Introduction

Nephrolithiasis is a common urologic disease and a major cause of morbidity spanning all age groups with a significant economic burden. The most prevalent are medium-sized renal stones (10–20 mm) while the best management option for these stones depends upon various factors including, stone size, location, density and calyceal anatomy [1, 2].

During the past decade, several treatment options have been introduced and matured including PCNL, SWL and RIRS. PCNL is superior to SWL for stone clearance and is not impaired by anatomical factors and stone density. The evolution of SMP in recent years has further optimized effectiveness by decreasing the complications and morbidity of PCNL [3]. With the new generation of flexible ureterorenoscopes, RIRS has emerged as a favored treatment option for lower-volume renal stones. However, its effectiveness is significantly dependent on calyceal anatomy and stone density [4].

This study was undertaken to bridge the void in high-level evidence that currently exists comparing these two minimally invasive treatment modalities in the treatment of renal calculi ≤ 2 cm. The primary objective was to compare short-term stone-clearance rates. The secondary objectives included outcomes in operating time, hemoglobin (Hb) decline, Clavien–Dindo complication rates, postoperative pain and hospital stay.

## Patients and methods

This prospective, inter-institutional, observational and propensity matched study was conducted from September 2018 to April 2019. The study was performed in two high-volume tertiary referral centers, performing > 2000 procedures for stone disease per year including PNLs, Ureteroscopy, and RIRS cases. In each team, two experienced faculty performed the procedures. From data of 593 patients with PCNL and 239 patients with RIRS, 149 patients were included. 75 patients undergoing SMPs for renal calculi of ≤ 2 cm (Group 1) were matched with 74 patients undergoing RIRS (Group 2). The matching was done for stone size, BMI, gender. The study was approved by the Institutional Review Board at both Institutions and informed patient consent was obtained from all study participants (IEC 532/2018 and IRB board approval number– EC/117/2010).

**Figure:**
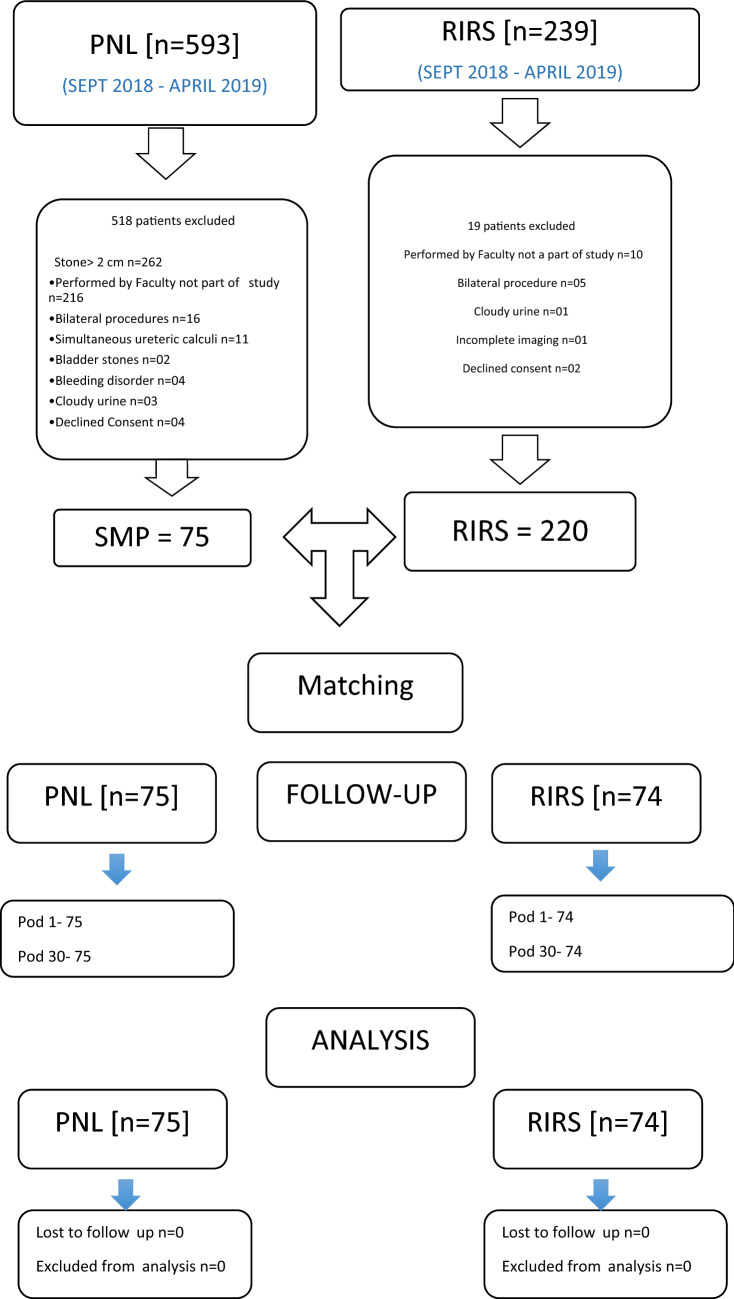

The inclusion criterion was a single renal stone of maximum diameter of ≤ 2 cm or multiple stones with a maximum cumulative diameter of ≤ 2 cm in the same calyx not needing another puncture. The stone size was defined as the maximum diameter as determined by non-contrast CT. Exclusion criteria were patients aged < 18 years, uncorrected coagulopathy, on antiplatelets or anticoagulants, and active urinary tract infections.

Preoperative evaluation included full blood count, serum creatinine and electrolytes, urine culture, coagulation profile, renal ultrasonography (US), X-ray of the kidneys, ureter and bladder (KUB), and non-contrast CT. All procedures were performed under general anesthesia. One dose of antibiotic on induction, followed by two doses postoperatively was given.

### SMP

A 5-Fr ureteric catheter was placed retrograde, and patient positioned prone.

The selected calyx was accessed by the urologist under fluoroscopic guidance using an 18G needle and a 0.032-inch guide wire. A single-step metal dilator was then passed, over which the SMP sheath was advanced into the system.

The 14-Fr SMP sheath has an internal working channel caliber of 12.5 F and an oblique channel to which suction is attached (first-generation SMP; R.K. Medical devices, Mumbai, India). This oblique part consists of a pressure vent controlled by its degree of occlusion by the surgeon’s thumb. The irrigation fluid is connected to the side port of either the Nephroscope or SMP sheath. A 12-/7.5-F Nephroscope (Richard Wolf, Knittlingen, Germany) is used. The stone was either completely fragmented or dusted using a Holmium YAG laser. The resultant dust and tiny stone particles pass through the oblique channel into the suction collection bottle.

The larger hard fragments were extracted using a 3-Fr grasper. A JJ stent was inserted whenever indicated. The mean operative time was calculated as the time taken from the calyceal puncture to the completion of the procedure with a skin suture. Immediate stone clearance was confirmed by nephroscopy and fluoroscopy.

### Retrograde intra-renal surgery

In lithotomy position, a ureteral guide wire was advanced followed by insertion of an 11/13 or 9/11 Fr ureteral access sheath (Cook Medicals, Bloomington, United states). Tight ureters were stented for a subsequent staged procedure. A Flex X2 ureterorenoscope (Karl Storz, Tuttlingen, Germany) was used in combination with a Holmium Yag Laser for stone fragmentation or dusting. Clear vision was maintained by continuous flow of normal saline irrigation at height of 40 cm attached to the flexible ureteroscope. During stone fragmentation, the flow was adjusted to keep excessive movement of stone as well as clear vision. The laser settings were dependent on the stone density and stone size. Indications for post-operative stenting included the presence of infection, pelvicalyceal system injury, access sheath-related ureteric mucosal injury, preoperative renal impairment, a solitary renal unit, a large stone burden and prolonged operative time. The mean operative time was calculated as the time taken from cystoscopy to stone clearance.

In either of the procedures, if only a JJ stent is placed, the procedure was considered “Tubeless” and when neither JJ stent nor PCN were placed the procedure was termed “Totally Tubeless”. In both groups, postoperative flank pain was assessed using the Visual Analog Scale (VAS) score. Parenteral Tramadol on demand was administered as per institutional protocol in first few hours and orally later when needed. The Clavien–Dindo classification was used to classify the complications. Plain X-ray KUB and renal US were performed by a radiologist to assess stone clearance on the first postoperative day and repeated at 1 month, at the proposed time of stent removal. Any visible hyperechoic area with posterior acoustic shadow on USG and/or any radiopacity on Xray KUB was considered as not stone-free.

### Statistical analysis

The sample size was calculated using PASS software (NCSS, LLC, East Kaysville, UT, USA), Chi-square test based formula for Confidence Intervals for the difference between two proportions was used with a 80% power, 5% alpha and 95% level of confidence to demonstrate a difference within 12% in SFR (98% vs 85%) between SMP and RIRS after 3 month of surgery, the minimum sample size for each group was estimated to be 68. To account for patients lost to follow-up and study withdrawals, this number was increased to 75.

Propensity-score matching was done using XLSTAT 2020.3.1.1005 software utilizing Two-sample *t *test and *z*-test; and tests on contingency tables (Chi-square).

## Results

**Fig. 1:**
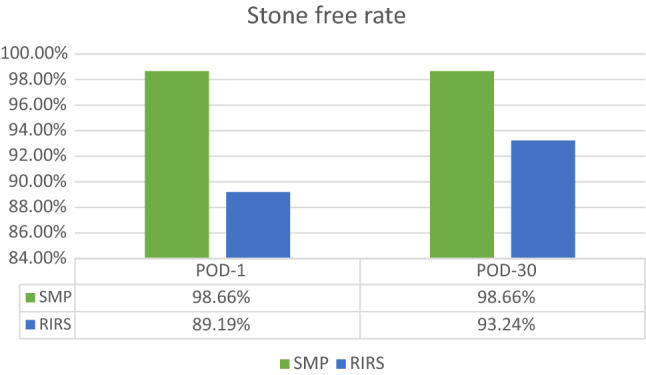
Comparison of stone-free rate in the two study groups

75 patients underwent SMP (Group 1) and 74 patients underwent RIRS (Group 2). The patient demographics and stone characteristics of both groups were propensity matched (Table 1). The stone-free rate (SFR) was statistically significantly higher in Group 1 on POD-1 (98.66% vs 89.19%, *p* = 0.015), and higher but not statistically significant on POD-30 (98.66% vs 93.24%, *p* = 0.092) (Fig. 1). The mean (SD) operative time was significantly shorter in Group 1, at 36.43 (14.07) vs 51.15 (17.95) min (*p* < 0.0001). Total number of patients with totally tubeless procedure was significantly higher in Group 1 compared to Group 2 (28 vs 6, *p* < 0.0001).

**Table 1:** Demographic and stone characteristics in the two study groups

	SMP	RIRS	*p* value
No. of renal units	75	74	
Gender distribution (male: female)	57: 18	51: 23	= 0.333
Mean age (years)	48.36 (19–76)	48.56 (23–76)	= 0.925
Mean BMI (kg/m^2^)	25.23	25.62	= 0.544
Comorbidities			
Diabetes mellitus	8	11	= 0.442
Hypertension	16	27	= 0.041
Ischemic heart disease	8	5	= 0.383
Mean size of stone (cm^2^) (SD)	1.48 (0.78)	1.41 (0.35)	= 0.477
Mean Hounsfield units (SD)	1247 (191)	1012 (327)	< 0.001
Laterality (right: left)	38: 37	35: 39	= 0.681
Site of stone			= 0.954
Pelvis	29	26	= 0.655
Upper calyx	9	10	= 0.708
Middle calyx	16	15	= 0.873
Lower calyx	21	23	= 0.759
Mean Pre-op serum creatinine (mg/dl)	1.09 ± 0.44	1.13 ± 0.60	= 0.624

The mean hemoglobin drop was significantly less in Group 1, at 0.31 vs 0.53 gm% (*p* = 0.020). The mean increase in post-operative serum creatinine was similar in the two groups (*p* = 0.595).

There were more Clavien–Dindo complications in Group 2 as compared to Group 1 (Table 2). The mean VAS pain score was significantly less in Group 2 at 6 and 12 h postoperatively (2.51 vs 3.67, 1.85 vs 2.40, respectively; *p* < 0.0001), whereas mean VAS pain score was significantly less in Group 1 at 24 h postoperatively (0.31 vs 1.01, *p* < 0.0001). The mean hospital stay was also significantly shorter in Group 1 (28.37 vs 45.70 h, *p* < 0.0001).

**Table 2:** Intra-operative and postoperative variables in the two study groups

	SMP	RIRS	*p* value
Mean operative time minutes (mean ± SD)	36.43 ± 14.07	51.15 ± 17.95	< 0.0001
Tubeless	75	74	
Totally tubeless	28	6	< 0.0001
Postoperative complications			
Clavien–Dindo Grade I	1	1	= 0.021
Grade 2	0	7	
Grade 3	0	2	
Stone-free rate			
POD–1	98.66%	89.19%	= 0.015
Pelvis	96.55 (1)	88.46 (4)	= 0.253
Upper calyx	100	100	
Middle calyx	100	93.33 (3)	= 0.301
Lower calyx	100	82.61 (4)	= 0.047
POD-30	98.66%	93.24%	= 0.092
Pelvis	96.55 (1)	92.31 (2)	= 0.493
Upper calyx	100	100	
Middle calyx	100	93.33 (1)	= 0.301
Lower calyx	100	91.30 (2)	= 0.171
Mean hemoglobin decline (g %)	0.31 ± 0.49	0.53 ± 0.64	= 0.020
Mean post-op serum creatinine (mg/dl)	1.10 ± 0.35	1.14 ± 0.61	= 0.625
Mean increase in serum creatinine (mg/dl)	0.06 ± 0.09	0.07 ± 0.17	= 0.595
Mean pain visual analog score			
1 h	3.67 (0.50)	2.51 (0.97)	< 0.0001
6 h	2.40 (0.49)	1.85 (0.77)	< 0.0001
24 h	0.31 (0.46)	1.01 (0.77)	< 0.0001
Hospital stay (h)	28.37 ± 3.6	45.70 ± 16.7	< 0.0001

## Discussion

It is generally accepted that the best treatment for renal stones < 2 cm is ill-defined and undergoing continuous debate [2–11]. According to the European Association of Urology (EAU) guidelines, shockwave lithotripsy (SWL), percutaneous Nephrolithotomy (PNL), and retrograde intrarenal surgery (RIRS) are recommended treatment options for the treatment of renal calculi < 2 cm [5].

The present study shows that SMP in patients with renal stones of up to 2 cm has higher SFRs, in addition to lower complication rates, blood loss and hospital stay when compared to RIRS. This innovation provides good efficacy in a single session combined with reduced morbidity and most probably economy.

Parallel innovations in both miniaturization of PNL, in an attempt to decrease morbidity [3–9], and digital imaging alongside fiber-optic technology in FURS [4, 11], have all added to the urologist’s armamentarium, and are competing to achieve this much coveted position in the treatment algorithm of such stones.

The last decade has seen numerous studies comparing PNL to RIRS in the management of renal calculi < 2 cm (Table 3). While PNL and RIRS have extensively been analyzed in systematic reviews and meta-analysis [12–17], only one study to date has reviewed SMP versus RIRS for renal stones < 2 cm [18].

**Table 3:** Comparison studies of PNL versus RIRS

Study	Design	Stone	Modality	Cases—*N*	Stone size (mm)	Months follow-up	SFR (%)	SFR definition	
Zhang [29]	RCT	LPS 1-2 cm	UMP FURS SWL	60 60 60		3	98 92 73	Fragment < 3 mm	16.67 8.33 6.67
Zeng [18]	RCT	LPS 1-2 cm	RIRS SMP	80 80	14.3 ± 3.4 15.0 ± 2.9	3	86.8 97.4	Fragment < 3 mm	7 7
Kandemir [30]	RCT	LPS ≤ 1.5 cm	RIRS PNL-4.85Fr	30 30	11.5 10.6	3	86.7 83.3	No residual stone	16.7 20
Bozzini [31]	RCT	LPS < 2 cm	SWL RIRS PNL	194 207 181	13.8 ± 3.1 14.8 ± 2.7 15.2 ± 3.3	3	61.8 82.1 87.3	Asymptomatic fragment < 3 mm	6.7 14.5 19.3
Fayad [32]	RCT	LPS < 2 cm	RIRS PNL-16Fr	60 60	14.1 ± 3.0 14.7 ± 3.0	3	84.3 92.7	Fragment < 2 mm	3.3 5
Akbulut [33]	CCT-R	LPS < 2 cm	PNL-18Fr RIRS	31 63	137.4–62.5 mm^2^ 137.7–40.9 mm^2^	1	90.3 85.7	Asymptomatic fragment < 3 mm	29 8
Ozgor [34]	CCT-R	1–2 cm	PNL18-20Fr RIRS	56 56	19.5 ± 3.9 18.3 ± 3.2	1–3	80.4 76.7	Fragment < 2 mm	30.3 5.3
Demirbas [35]	RCT	1–2.5 cm	PNL-14Fr RIRS	30 43	185.86–88.29mm^2^ 181.70–114.18mm^2^	1	80 74.4	Fragment < 3 mm	23.3 13.9
Kumar [36]	RCT	LPS 1–2 cm	PNL-18Fr RIRS SWL	41 42 43	13.3 ± 1.3 13.1 ± 1.1 13.2 ± 1.2	3	95.1 86.1 73.8	Fragment < 4 mm	24.3 9.3 7.1
Lee [37]	RCT	> 1 cm	PNL-18Fr RIRS	35 33	39.1 ± 30.7 28.9 ± 17.5	3	85.7 97	Fragment < 2 mm	25.7 27
Schoenthaler [38]	CCT-R-(match)	1–2 cm	PNL-14Fr RIRS	30 30	15.1 14.4	–	84 87	–	7 7
Ramon de Fata [39]	CCT	1–3 cm	PNL-4.85Fr RIRS	8 12	1.9 cm^2^ 1.3 cm^2^		87.5 91.7		12.5 8.3
Kirac [40]	CCT-R	< 1.5 cm	PNL ≤ 20Fr RIRS	37 36	1.05 ± 0.22 1.02 ± 0.29	1–3	89 88.9	Fragment < 3 mm	18.9 16.7
Pan [41]	CCT-R	2–3 cm	PNL-18Fr RIRS	59 56	22.37 ± 2.7 22.28 ± 2.6	1	96.6 71.4	Fragment < 2 mm	11.9 16.1
Kruck [42]	CCT-R		PNL-18Fr RIRS SWL	172 108 202	12.6 ± 9.5 6.8 ± 6.9 7.5 ± 5.1	3	79.6 77.8 58.4	No stones	11.5 8.3 5
Ozturk [43]	CCT-R	1–2 cm	PNL RIRS SWL	144 38 221	1.74 ± 0.15 1.73 ± 0.15 1.70 ± 0.16		93.7 73.7 –		13.2 5.3 3.2
Sabnis [26]	RCT	< 1.5 cm	PNL-4.85Fr RIRS	35 35	1.1 1.04	3	97.1 94.3	No residual stone	28.6 14.3
Resorlu [44]	CCT-R	1–2 cm	PNL RIRS SWL	140 46 251	17.3 ± 3.6 15.6 ± 3.4 14.9 ± 2.9	3	91.4 87 66.5	Fragment < 3 mm	22.1 10.9 7.6
Aboutaleb [45]	CCT-R	1–2 cm	PNL RIRS SWL	19 13 24	1.73 ± 0.33 1.45 ± 0.32 1.56 ± 0.43		89.5 84.6 –		31.6 46.2 41.7
Bozkurt [46]	CCT-R	1.5–2 cm	PNL RIRS	42 37	1.70 ± 0.12 1.65 ± 0.69	2	92.9 89.2	Fragment < 3 mm	16.7 18.9
Kuo [47]	RCT	LP < 2.5 cm	PNL RIRS	15 13		3	66.7 45.6		6.7 0

*CCT-R* case–control trial-retrospective

The unique irrigation-suction channel with the 14Fr sheath gives SMP a versatility unattained in the prior versions of PNL, as articulated by none other than Dr. Peter Alken, in his editorial, ‘based on my more than 40 years of experience with percutaneous stone removal and intense knowledge of the changes that were introduced I think it is justified to state the SMP technique is the most significant progress in this field and it will likely become the dominant method for percutaneous stone management in the future’ [6]. With miniaturized PCNL, from Micro-perc, Mini-perc to UMP, the reported SFRs have been in the range of 60–90% [3–9]. In comparison, the SFR in our present SMP group is higher at 98.6% [3–9]. This is attained by the dual modality of negative pressure suction, providing clear vision for effective fragmentation and extraction along with the use of a 3-F grasper to extract fragments from different calyces. Also, a lower intra-renal operating pressure ≤ 25 mmHg is better maintained [19].

On the other hand, RIRS has the distinct advantage of not breaching the renal parenchyma. The ‘trade-off’ is however its dependence on the anatomical favorability of the ureter and pelvicalyceal system. The lower calyceal stones are a particular challenge, especially those with acute IPAs and narrow infundibula. Prolonged lasing time is yet another issue. The modern generation fURS with its enhanced maneuverability and vision affords a less invasive and safe option, but its effectiveness is tempered by the stone burden and density [20]. Studies in the management of “small renal stones”, show RIRS to have a stone-free rate of about 65–92% [4]. This is lesser in lower calyceal calculi, especially with unfavorable anatomy. Other drawbacks are the need for staged procedures in the case of hostile tight ureters, the higher costs of RIRS compared to PCNL, the risk of ureter injury, and the requirement of post-operative JJ stent [21, 22].

Stone-free rates of SMP range from 93.2% to 96.2% [3, 6, 18, 23]. This is in line with the present study and significantly higher than RIRS on POD-1 (98.66% vs 89.19%, *p* = 0.015). In SMP, small fragments and dust are removed by suction. Larger fragments were removed using 3F grasper. In RIRS, complete extraction of all fragments was not possible intraoperatively, thereby necessitating ureteral stenting. Some fragments may settle in the lower calyx and possibly act as a nidus for future stone growth. Upon subgroup analysis of stone-free rates in lower calyceal stones, SMP is superior to RIRS both on POD 1 and POD 30 (Table 2). Difficult angulation, narrow infundibula and prolonged lasing time make complete stone clearance challenging [24]. SMP bypasses these anatomical restrictions and allows for the effective removal of all stone fragments in a single treatment session.

At 1-month follow-up, SFR was still better in the SMP group, but was not statistically significant anymore (98.66% vs 93.24%, *p* = 0.092). This increased SFR in RIRS at 1 month, is explained by the spontaneous passage of the smaller stone fragments. SMP gives early stone-free rates while in RIRS, SFR keep on improving with time. SFR in RIRS on POD 30, though not statistically different were still lower than SMP. This difference would have been more significant had propensity matching included HU of stones. This highlights importance of patient counseling about postoperative fluid intake to let stone dust drain clear of urinary tract. There is no uniformity in published literature regarding standard time of evaluation for SFR in post RIRS patients.

The morbidity is on multiple aspects in the disadvantage of RIRS. The mean operative time was significantly longer in the RIRS group, the postoperative complications were significantly more common in the RIRS group. Interestingly, the Hb drop was higher, and hospital stay longer in the RIRS group.

The mean operative time was significantly longer in the RIRS group, 36.43 vs 51.15 min (*p* < 0.0001). This results from more time required to access, stabilize, fragment and extract the calculi in RIRS. In SMP, the suction helps to aggregate fragments at the opening of the sheath, thereby aiding in faster lithotripsy. Also, harder, bigger fragments can be retrieved using a grasper. Studies have already shown RIRS requiring longer operative times in stones of size > 1 cm [20].

Postoperative complications were significantly more common in the RIRS group (*p* = 0.021). 10 of the 75 patients in the RIRS group developed complications. 8 patients had postoperative fever managed conservatively. 1 patient required aspiration of urinoma detected intraoperatively. These were graded according to Clavien–Dindo classification. Grade 1 complication was noted in one patient in each group. Seven patients had grade 2 and two patients had grade 3 complications, which included a stent blockage that mandated an early stent removal on POD 3 and the other was a fluid collection in the abdomen that mandated an aspiration believed to be ureteric injury related to UAS insertion in the RIRS group. None of the patients had higher grade complications in SMP group.

Postoperative fever was the most common complication of the patients treated by RIRS. The increased renal pelvic pressures from irrigation may cause pyelovenous and pyelolymphatic backflow, leading to these infectious or non-infectious complications [25]. Conversely, SMP, with the continuous negative pressure aspiration maintains the intrarenal pressure consistently below 25 mmHg [19].

Mean increase in post-operative serum creatinine was similar in the two groups (*p* = 0.595). Mean hemoglobin drop was more in the RIRS group (*p* = 0.020). RIRS is associated with less Hemoglobin drop as compared standard PCNL. However, this comparison is not available with UMP, SMP. Micro-PNL has been found to be associated with reduced blood loss though not slighter than RIRS [26]. Mean hemoglobin drop in RIRS group could be due to continuous minimal ooze from access sheath associated Grade 1 mucosal injuries, mucosal injuries by laser fiber during stone pulverization. Prolonged time of fragmentation especially of lower calyceal stones with continuous irrigation often masks blood loss. Severe bleeding and sepsis have also been reported in other studies [27]. Straight tract in SMP with an easy maneuverability of the nephroscope in the collecting system, ease in laser fragmentation of stones in any calyx and stone fragments extraction collectively lead to reduced loss and decreased Hb drop in SMP. None of the patients in either groups had blood transfusion in our study.

Postoperative pain was significantly less in the RIRS group at 1 and 6 h (2.51 vs 3.67, 1.85 vs 2.40, respectively). The prolonged operative time with increased intra-renal pressures causing the renal capsule to stretch and potential extravasation can lead to persistent pain. However, at 24 h, pain was significantly less in the SMP group (0.31 vs 1.01). Postoperative pain is seen to be associated with nephrostomy tubes, tract size and intercostal nerve injury. All patients in our SMP group were ‘tubeless’. The SMP group had more ‘totally tubeless’ patients (28 versus 6 in the RIRS group), which could also explain the observed difference [21]. The absence of a stent also obviated another hospital visit for its removal.

Length of hospital stay was calculated in hours, after the surgery. The hospital stay was significantly shorter in the SMP group (28.37 vs 45.70 h) and was similar to that reported by Shah et al. [28] (28.37 vs 31.53 h). Reduced pain scores at 24 h, reduced complications and decreased need of any external drainage could all be contributory.

The ultimate goal of achieving ‘stone-free status’ in a single session with minimal invasiveness, minimal complications, short treatment time, decreased recurrence risk, and decreased costs is central in developing our treatment strategy for renal calculous disease, more so in countries with limited resources.

## Limitations

Our study utilized X-ray KUB and renal US to evaluate stone clearance. These minimized the radiation exposure and were economically viable in all our patients. However, non-contrast CT would have given a more accurate assessment. Another limitation was the prevalence of lower calyceal calculi as the second-commonest location after pelvic calculi in both study groups. This could, in part, account for the poorer results in the RIRS cohort. Another limitation of this study was that propensity matching did not include HU of stones, which may have changed results negatively for RIRS in terms of SFR on POD 30 and mean procedure time.

While institutional protocols were followed, some differences between both institutes may have influenced outcomes and complications. Although only high-volume surgeons have been involved in treating all patients, this may also not translate in generalizability of this study. Hence higher complications by a certain procedure may be reflected in the difference in techniques between operators. Obviously high volume does not automatically imply better outcomes per se. As for the evaluation, two different radiologists were involved in the study and different machines/equipment were used for evaluation. This may also result in differences in evaluation of SFR. Finally, VAS score was only for flank pain and USSQ stent-related questionnaire for stent-related symptoms was not used for patients with ureteral stents.

## Conclusion

SMP provides early stone-free rates as compared or better to RIRS in a single session combined with reduced morbidity, in the management of renal calculus of size less than 2 cm. Although SMP is associated with more early post-operative pain, it has significantly lower operative times, complication rates and a shorter hospital stay.

## Data Availability

Not applicable.
